# Surfactant-Free Decellularization of Porcine Auricular Cartilage Using Liquefied Dimethyl Ether and DNase

**DOI:** 10.3390/ma16083172

**Published:** 2023-04-18

**Authors:** Hideki Kanda, Kento Oya, Wahyudiono, Motonobu Goto

**Affiliations:** Department of Materials Process Engineering, Nagoya University, Nagoya 464-8603, Japan

**Keywords:** decellularization, extraction, liquefied gas, scaffold, subcritical fluid

## Abstract

The most common decellularization method involves lipid removal using surfactant sodium dodecyl sulfate (SDS) and DNA fragmentation using DNase, and is associated with residual SDS. We previously proposed a decellularization method for the porcine aorta and ostrich carotid artery using liquefied dimethyl ether (DME), which is free from the concerns associated with SDS residues, instead of SDS. In this study, the DME + DNase method was tested on crushed porcine auricular cartilage tissues. Unlike with the porcine aorta and the ostrich carotid artery, it is important to degas the porcine auricular cartilage using an aspirator before DNA fragmentation. Although approximately 90% of the lipids were removed using this method, approximately 2/3 of the water was removed, resulting in a temporary Schiff base reaction. The amount of residual DNA in the tissue was approximately 27 ng/mg dry weight, which is lower than the regulatory value of 50 ng/mg dry weight. Hematoxylin and eosin staining confirmed that cell nuclei were removed from the tissue. Residual DNA fragment length assessment by electrophoresis confirmed that the residual DNA was fragmented to less than 100 bp, which was lower than the regulatory limit of 200 bp. By contrast, in the uncrushed sample, only the surface was decellularized. Thus, although limited to a sample size of approximately 1 mm, liquefied DME can be used to decellularize porcine auricular cartilage. Thus, liquefied DME, with its low persistence and high lipid removal capacity, is an effective alternative to SDS.

## 1. Introduction

Cartilage has a high water content and a rich extracellular matrix that contains proteoglycans and type II collagen [1]. The cartilage is responsible for smooth joint movements, such as lubrication and shock absorption under loads. Cartilage has a layered structure that is durable under pressure [2], but vulnerable to rubbing movements such as shear forces. Furthermore, it is thought to be difficult for cartilage to heal spontaneously [3] because it has no nerves, nutritional blood vessels, lymphatic vessels, or cellular components. Therefore, cartilage tissue transplantation is an important treatment option. In the case of reconstructive surgery of the nose and ears, transplantation of autologous tissue, such as one’s bone (ilium or skull) or cartilage (auricular, rib, or nasal septum cartilage), is free from rejection; however, in the long term, it is associated with the disadvantage of sacrificing the harvested part.

Porcine tissues support three-dimensional culture and are a common raw material for developing scaffolds suitable for human transplantation. Although many examples of aortic decellularization [4,5,6,7] exist, only a few reports have specifically reported auricular cartilage decellularization [8,9]. For bone, which is not cartilage but a similar tissue, a tortuous structure that simulates a realistic extracellular matrix structure will result in better cell adhesion than if the voids in its scaffold have relatively linear microchannels [10]. The porous structure of a scaffold is also important [11]. Therefore, if animal cartilage can be decellularized and its tortuous void structure can bemaintained, a scaffold with a high culture performance will likely be obtained. A few previous studies have demonstrated a decellularization procedure consisting of three main steps: lipid removal by sodium dodecyl sulfate (SDS), DNA fragmentation using Dnase treatment, and, finally, removal of fragmented DNAs by washing [9]. However, the method used by Kheir et al. [9] does not remove sufficient cells; therefore, a new method has been proposed wherein a washing step using the proteolytic enzyme elastase is incorporated between the SDS and DNase treatments [8]. However, the decellularized tissue is severely destroyed when the elastase concentration is incorrectly adjusted [8].

In addition to the inadequate decellularization ability of SDS, another concern with SDS is its high affinity for proteins, which makes it toxic; repeated washing is required to completely remove SDS from the tissue [12]. Notably, the tissue was irreversibly damaged even when the SDS was removed. In previous studies pertaining to porcine aorta decellularization with SDS and DNase, the gaps between collagen fibers widened, as confirmed by hematoxylin and eosin staining [6]. Owing to the irreversible damage caused by SDS, decellularized tissue treated with SDS expands more than the original tissue, and its tensile strength is reduced to approximately half that of the original tissue [4]. Therefore, decellularization methods that do not involve using SDS have been investigated. For example, agents such as peracetic acid and techniques such as alkaline swelling have been reported [13]. When using peracetic acid, thin tissues such as the small intestinal submucosa [14] and bladder tissue [15] can be decellularized; however, the cells are not completely removed [14]. Furthermore, the mechanical properties of decellularized tissues are altered, resulting in increased yield stress and elastic modulus [15]. In other words, the formation of a stiff extracellular matrix indicates that peracetic acid may not be suitable for tissues where the conservation of elasticity is required. Alkaline treatment also results in negatively charged swelling of collagen in the bovine pericardium, which reduces the glycosaminoglycan content and viscoelasticity of the tissue [16]. Based on the idea that tissue damage and residue caused by chemical substances are fundamental problems, some approaches have attempted to treat substances that cause little tissue damage and are so volatile that they do not persist, or are not problematic even if they persist. Representative examples include supercritical carbon dioxide and ultrahigh-pressure water. However, the supercritical carbon dioxide method requires ethanol [6] or limonene [17] to be mixed with the supercritical carbon dioxide because supercritical carbon dioxide alone cannot remove cell membranes. Another limitation is that the operating pressure of this method is 15 MPa, which requires extremely expensive equipment. In other studies, a high hydrostatic pressure technique was successfully used to decellularize carotid tissue, and SDS was not used in this case. However, this technique requires special processing equipment that can handle an ultrahigh pressure of 980 MPa (abs) [7,18,19,20]. However, these techniques have not been applied to decellularizing auricular cartilage.

As a new approach for decellularization without SDS, a method for removing lipids from tissues using liquefied dimethyl ether (DME) as the solvent instead of SDS was proposed in 2021. DNA degradation by DNase and washing after lipid removal allow decellularization of the porcine aorta [5] and ostrich carotid arteries [21]. This method fulfilled all three conditions considered prerequisites for successful decellularization. First, no cell nuclei were observed upon microscopic examination of the hematoxylin–eosin-stained decellularized tissue. Secondly, the residual DNA in the decellularized tissue was less than 50 ng/mg dry weight, and the residual DNA fragments in the decellularized tissue were less than 200 bp [22,23].

DME is a solvent frequently used for the extraction of organic matter; for example, extraction from rice bran [24], river sediment [25], sugarcane leaves [26], hop [27,28], microalgae [29], livestock waste [30], soy beans [31], milk [32], and plants [33] has been demonstrated. The ability to handle high-moisture objects stems from the weak hydrogen bonds in DME [34], which allow liquefied DME to partially mix with water [35,36,37]. Miscibility with water is an important aspect in handling animal tissues with high water content. In addition to being water-soluble, the chemical and physical properties of DME differ from those of ethyl ethers. DME has a very low standard boiling point of −24.8 °C [38]. Therefore, when used as an extraction solvent, it must be pressurized above the saturated vapor pressure. Its low boiling point also means that it leaves little residue in the extract or the extraction residue [5,21]. The European Union permits its use as an extraction medium in a number of cases [39]. DME has also been designated as “Generally Recognized as Safe (GRAS)” by the Food and Drug Administration of the USA [40]. Bioassays have confirmed that DME dissolved in water is not toxic to indigenous bacteria present on the human skin [5]. Furthermore, unlike other alkyl ethers, the autoxidation of DME is comparable to that of liquefied petroleum gas (LPG) and it is safe enough to handle using routine LPG handling techniques [41]. Therefore, treatment with liquefied DME can be performed at room temperature to protect the material from thermal damage. This may provide additional advantages for applications in pharmaceutical and food industries. One such application is decellularization using liquefied DME.

Although liquefied DME has an excellent extraction ability and safety profile, it can extract water, which is problematic during tissue decellularization. During the decellularization of the porcine aorta, C=N bonds are temporarily formed in the collagen fibers of the porcine aorta owing to the Schiff base reaction associated with water extraction [5]. These C=N bonds are degraded by aqueous DNase treatment after lipid extraction with liquefied DME [5]; however, the elasticity of the decellularized tissue in the porcine aorta is reduced [4]. In the case of the ostrich carotid arteries, temporary C=N bonds were not formed [21]. On the other hand, this C=N bond was not formed in the ostrich carotid artery, and the elasticity of the ostrich carotid artery was not reduced at all [42]. This difference may be attributed to the presence or absence of the Schiff base reaction induced by liquefied DME treatment. However, owing to the paucity of experimental examples, it is also unclear in which species and in which tissues this chemical change occurs; consequently, the causal relationship between this chemical change and the mechanical change is also unclear. Therefore, performing decellularization tests on other samples is necessary to gain fundamental knowledge.

**Figure 1:**
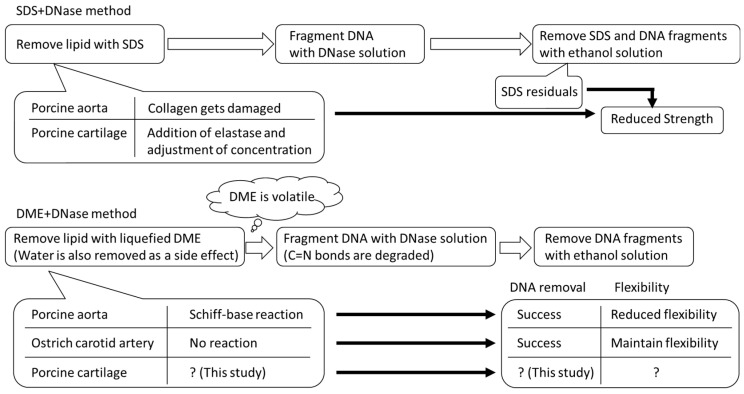
Decellularization procedure of this study using SDS and liquefied DME.

The conditions underlying the temporary formation of these C=N bonds need to be studied further using more samples. In addition, when targeting the auricular cartilage for decellularization, although elasticity is not as important as it is for the arteries, decellularization itself is difficult. Therefore, in this study, we applied a decellularization technique that combines lipid extraction with liquid DME and DNA fragmentation using a DNase solution on porcine auricular cartilage. Figure 1 illustrates the decellularization procedures using the conventional SDS and liquefied DME that have been described so far, the problems and advantages of each, and the position of this study. This study provides information on how to achieve the decellularization of cartilage tissue, which is considered difficult to decellularize without SDS, and how to explore the conditions under which the Schiff base reaction occurs in collagen using this new method with liquefied DME.

## 2. Materials and Methods

### 2.1. Materials

**Figure 2:**
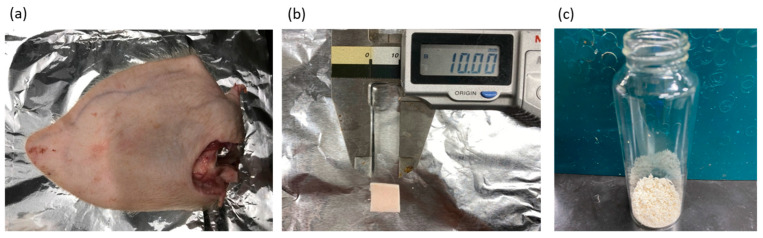
(**a**) Pig ear before removing auricular cartilage, (**b**) 10 × 10 × 1 mm sample, (**c**) 710–1000 μm-sized sample.

Fresh porcine auricular cartilage was obtained from a local slaughterhouse (Tokyo Shibaura Organ Co., Ltd., Tokyo, Japan). The porcine auricular cartilage used in this study was not slaughtered specifically for this experiment but from pigs slaughtered for meat processing. The water content of the cartilage was 43.5 ± 0.2 wt%, which was determined by the weight difference before and after heating at 107 °C until the weight remained constant using a moisture meter (Frontlab, AS ONE Corporation, Tokyo, Japan). Drying at 107 °C is a condition that can define the total water content of coal, including both free and hydrogen-bonded water, according to ISO 579 [43]. The intention of drying at 107 °C as required by ISO 579 is to desorb water by cleaving hydrogen bonds between the water and hydrophilic groups such as -OH and -COOH, which are present in large amounts in peat of plant origin. Many similar hydrophilic groups were present in the samples used in this study, and to desorb water from these hydrophilic group, drying per ISO 579 was employed. The moisture content of the peat close to the biomass was also specified in this method; therefore, we followed these conditions. Large amounts of excess lipids from the wet auricular cartilage tissues were trimmed using a knife. As discussed in detail in the Results section, the decellularization of porcine auricular cartilage depends on the original tissue’s size. For this reason, two types of experiments were conducted: one in which the porcine auricular cartilage (Figure 2a) was cut with scissors into sheets of 10 mm × 10 mm × original thickness (~1 mm) (Figure 2b), and the other in which the cartilage was crushed using a food mill (Mini Blender ECG62, Melitta Japan, Minden, Germany). The crushed porcine auricular cartilage was sieved to obtain a uniform particle size of 710–1000 μm (Figure 2c).

The total lipid content in the porcine auricular cartilage was determined using the Bligh–Dyer method [44]. Briefly, the cartilage was soaked in an extract solution containing methanol/chloroform/water = 2/1/0.8 (*v*/*v*/*v*) and stirred for three days. After filtration, the filtrate was evaporated using an evaporator (N-1200A; ULVAC Kiko. Inc, Miyazaki, Japan). The lipid content among different pigs varied greatly, between 5.1% and 16.3% per dry weight of porcine auricular cartilage. Therefore, when examining the amount of lipid removed with liquefied DME, the lipid content and the amount of supplied liquefied DME were evaluated as a ratio to the total lipid content defined by Bligh–Dyer extraction for the porcine auricular cartilage obtained from the same pig. A DNase solution (0.2%, DNase I; Grade II, from bovine pancreas; 10104159001, Roche Diagnostics, Tokyo, Japan) was used to fragment the porcine DNA. HPLC-grade methanol, chloroform, and water were purchased from FujiFilm Wako Pure Chemical Corporation (Osaka, Japan). The reagents and equipment not listed here are the same as those used in previous studies [5,21].

### 2.2. Decellularization by Liquefied DME and DNase

The decellularization protocol combining liquefied DME and DNase consisted of three steps:

Lipid extraction using liquefied DME

DNA fragmentation using DNase solution

Removal of fragmented DNA using ethanol solution

The difference between this method and conventional decellularization that the use of liquefied DME instead of SDS in the first step. In addition, degassing was performed as a pre-treatment for DNA fragmentation using a DNase solution. We repeated all the steps of the decellularization protocol three times under each condition and analyzed the obtained samples three times to verify their reproducibility.

#### 2.2.1. Lipid Extraction by Liquefied DME

The equipment and protocol for lipid extraction using liquefied DME were the same as those previously reported, with minor differences [5,21]. The only marginal differences are shown below, and the parts that are not mentioned are the same as those previously reported [5,21]. A total of 8.17 ± 0.03 g (10 × 10 mm × original thickness) or 8.04 ± 0.05 g (crushed) of the wet porcine auricular cartilage tissues were loaded into the extraction column reported in the previous reports [5,21]. When 60 mL of DME was stored in the vessel, the manual flow control valve was stopped and the vessel was quickly replaced with a new vessel.

#### 2.2.2. DNA Fragmentation Using DNase Solution

In the protocol used for the DNA fragmentation of DNA, the DNase solution differed from the previously reported decellularization protocols [5,21]. The treated tissue was immersed in a DNase solution and depressurized using an aspirator for 15 min to eliminate air bubbles. These bubbles indicate the presence of air that entered the porous structure of the tissue after treatment with liquefied DME. DNase treatment after this step was performed as described previously [5,21]. The maximum concussion time of the porcine auricular cartilage in the DNase-saline solution was 10 days.

#### 2.2.3. Removal of Fragmented DNA Using Ethanol Solution

Fragmented DNA was removed using ethanol solution as previously described [5,21].

### 2.3. Analysis of the Treated Tissue

The treated tissues were analyzed as described previously [5,21]. The analysis included the following: (1) hematoxylin–eosin staining to observe the removal of cell nuclei; (2) UV–Vis measurements after the degradation step using proteolytic enzymes to quantify residual DNA; (3) determination of DNA fragment distributions using agarose gel electrophoresis; and (4) analysis of Fourier-transform infrared spectroscopy (FT-IR) spectra to investigate the cross-linked structure of the DME-extracted porcine auricular cartilage and the decellularized tissue finally obtained. The amount of DME remaining in the decellularized tissue was quantified using GC-MS according to the method described in a previous report [5]; however, DME was not detected in any of the samples.

## 3. Results and Discussion

### 3.1. Lipid and Water Extraction Using Liquefied DME

**Figure 3:**
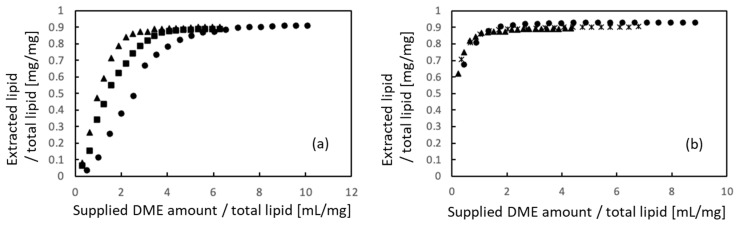
Lipid extraction from porcine auricular cartilage using liquefied DME. (**a**) 10 × 10 × 1 mm samples, (**b**) 710–1000 μm-sized samples. The three symbols represent individual differences among three samples.

Figure 3a shows the efficiency of lipid extraction from porcine auricular cartilage using liquefied DME for samples cut into 10 × 10 × 1 mm pieces and Figure 3b shows the results obtained for samples crushed to 710–1000 μm. Because the total lipid content of the untreated samples differed in each case, the amounts of extracted lipids and supplied liquefied DME were normalized using the total lipid amount, which was determined using the Bligh–Dyer extraction method [44]. In the initial stage of DME extraction, the 10 × 10 × 1 mm samples required more liquid DME to extract the lipids than that required by the 710–1000 µm-sized samples. This difference was due to the diffusion rate of liquefied DME but did not affect the main outcomes of this study. Finally, 89.0–90.9% of the total lipids were extracted from the 10 × 10 × 1 mm samples, whereas 90.1–93.0% of the total lipids were extracted from the 710 to 1000 µm-sized samples. The final amount of extracted lipids was similar for both samples, suggesting that liquefied DME penetrated deep into the tissues. In a previous study on the extraction of lipids from microalgae using liquefied DME [27], the original microalgae before extraction contained a large amount of nitrogen due to protein, but the extracted lipids contained less nitrogen despite their dark green color, suggesting that the origin of the nitrogen in the lipids was not protein but chlorophyll. Furthermore, in a previous study on the extraction and removal of lipids and water from livestock waste, the ratios of carbon and hydrogen in the extracted lipids reached 75.40 wt% and 11.81 wt%, respectively, whereas the ratio of nitrogen was only 0.87 wt%, indicating that little protein was extracted using liquefied DME [30]. Thus, almost all proteins remained in the cartilage tissue.

**Figure 4:**
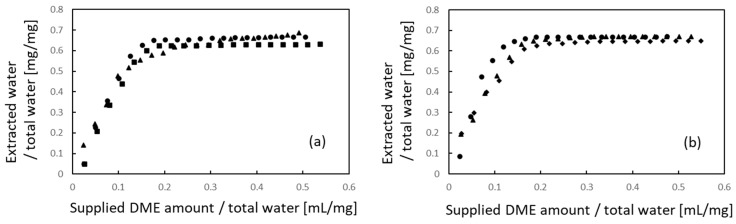
Water extraction from porcine auricular cartilage using liquefied DME. (**a**) 10 × 10 × 1 mm samples, (**b**) 710–1000 μm-sized samples. The three symbols represent individual differences among three samples.

Figure 4 shows the extraction behavior of water. Similar to the lipids, the amounts of water extracted and liquefied DME supplied were normalized to the total water content. When comparing Figure 4a,b, no apparent difference in either the initial or final extraction volume with respect to the sample size was observed in the case of water. In the case of the 10 mm × 10 mm × 1 mm samples, 63.0–68.6% of the total water was removed. In the case of 710–1000 µm samples, 64.7–67.1% of the total water was removed. Thus, the porcine auricular cartilage samples were not completely dried using liquefied DME, and some water remained.

This is in contrast to a previous study, wherein when this method was applied to the porcine aorta [5], 90.1% of the water held by the sample was extracted, leaving the aorta completely dry. It also differs from previous studies in which lipids and functional components were extracted from river sediment [25], biosolids [30], microalgae [29], and plants [33], but were completely dried. This implies that the water-holding capacity of protein-based porcine auricular cartilage is higher than that of the porcine aorta and plants. Because such a strong water-holding effect has not been observed in previous studies of decellularization in the porcine aorta [5] and ostrich carotid artery [21], it is inferred that the molecular interaction between water and proteoglycan, which constitute cartilage, is stronger than that between water and collagen and elastin, which constitute vessels.

### 3.2. Hematoxylin–Eosin Staining

**Figure 5:**
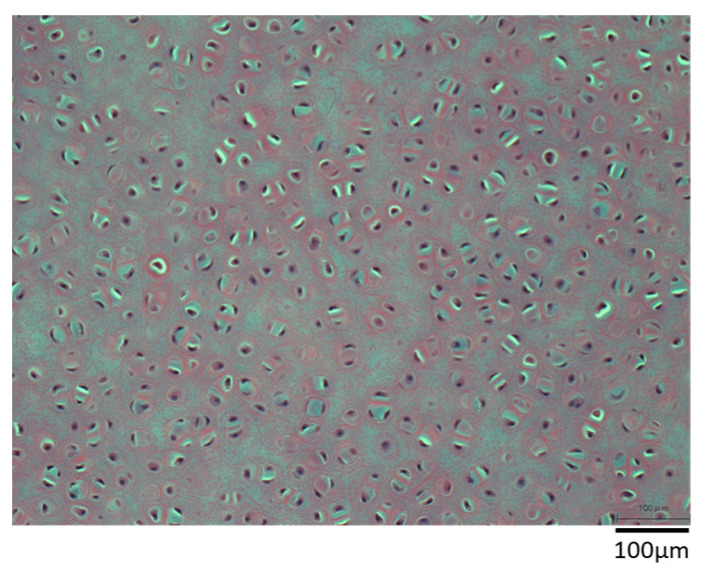
Hematoxylin–eosin staining of 10 × 10 × 1 mm sample. DNase treatment was applied for 7 days without degassing as a pretreatment.

Figure 5 shows a sample of 10 × 10 × 1 mm porcine auricular cartilage removed from lipids using liquefied DME and immersed in a DNase solution for 7 days without degassing. Nuclei were clearly visible, suggesting that the DNA was not removed. In addition, some parts of the image appear brighter than others. It was speculated that the lipid- and water-removed porcine auricular cartilage had a cavity where the lipid and water had been removed, and that the air in the cavity prevented the DNase solution from entering the interior of the porcine auricular cartilage. Therefore, subsequent tests were degassed prior to DNase solution treatment, as described in the experimental section. This phenomenon of inhibition of the permeation of aqueous DNase solution was observed for the first time in this study and has not been observed in previous studies on the decellularization of porcine aorta [5] or ostrich carotid arteries [21].

**Figure 6:**
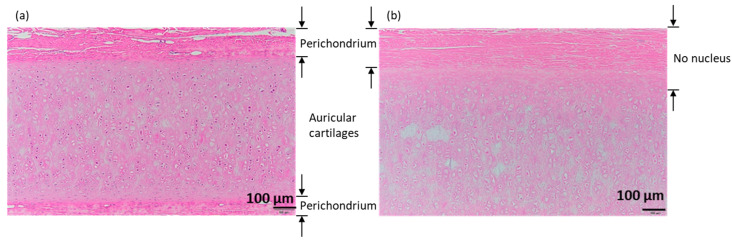
Hematoxylin–eosin staining of 10 × 10 × 1 mm samples. (**a**) Untreated. (**b**) DME extraction followed by DNase treatment for 7 days with degassing. The images shown below are enlarged images of the area enclosed by the rectangle in the upper right corner of the images shown above.

The results obtained from hematoxylin–eosin staining of porcine auricular cartilage cut into 10 × 10 × 1 mm samples are shown in Figure 6 for (a) untreated samples and (b) samples decellularized by lipid extraction using liquefied DME, followed by degassing and DNase treatment for 7 days. The perichondrium was observed above and below the untreated and decellularized samples. In the untreated group, several purple-stained cell nuclei were observed in the perichondrium and cartilage tissues. In contrast, in decellularized samples, few cell nuclei were identified in the perichondrium and in a portion of the auricular cartilage tissue at a depth of approximately 100 μm from the perichondrium at the boundary. However, in the central portion of the auricular cartilage, circular materials, such as cell nuclei which was at a depth of more than 100 μm, were visible, albeit lighter in color. This coloration is comparable to that found in the bovine cartilage that was decellularized by SDS and DNase treatment followed by washing with proteolytic enzyme elastase in a previous study [8] and is expected to be sufficiently decellularized; however, this observation alone was not considered to be conclusive.

**Figure 7:**
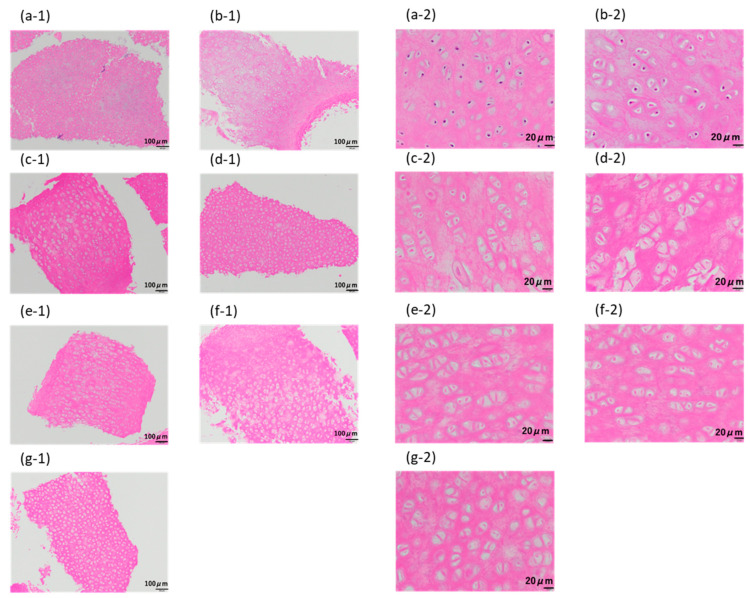
Hematoxylin–eosin staining of 710–1000 μm-sized samples. (**a**) Untreated. (**b**) After lipid extraction using liquefied DME. (**c**–**g**) DNase treatment for (**c**) 1, (**d**) 3, (**e**) 5, (**f**) 7, and (**g**) 10 days following the DME extraction. The images designated by “-1” indicate the original images, while those designated by “-2” indicate the enlarged images.

As shown in Figure 7a, for the samples crushed to 710–1000 µm, the purple-stained cell nuclei were clearly visible in the untreated samples. In the case of samples for which only the lipid extraction step was performed using liquefied DME, the color of the cell nuclei did not become lighter (Figure 7b). Compared with the porcine auricular cartilage samples, purple-stained cell nuclei were not visible in the samples washed in DNase aqueous solution for 1–10 days by immersion after the lipid extraction step (Figure 7c–g). However, the color of the observed circular substances, thought to be reminiscent of the cell nucleus, was comparable to that of the surrounding proteins. Even with the crushed samples, it was difficult to determine whether these observed circular components were cell nuclei; therefore, the success of decellularization was confirmed by quantifying the amount of remaining DNA. In the conventional method using SDS, for example, in the case of blood vessels, elastin fibers are charged by SDS, which causes the elastin fibers to straighten and widens the space between the fibers, resulting in a significant decrease in tissue strength [7]. Spreading between collagen fibers was not observed using this method.

### 3.3. Quantification of Residual DNA

**Figure 8:**
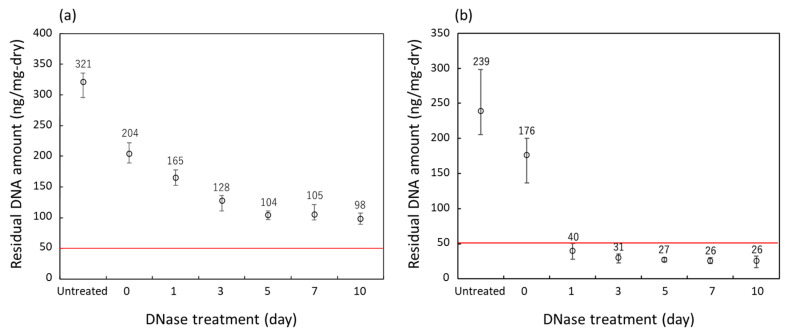
Residual DNA amounts in the porcine auricular cartilage upon treatment with liquefied DME. (**a**) 10 × 10 × 1 mm samples, (**b**) 710–1000 μm-sized samples. The DNase treatment for 0 days indicates that only lipid extraction with liquefied DME was carried out and no DNase treatment was performed. The red line indicates the regulated value.

The amount of DNA remaining in the samples obtained from the decellularization at various DNase treatment times of two groups of porcine auricular cartilage samples of different sizes is shown in Figure 8. Untreated samples and those which underwent lipid extraction using only liquefied DME are shown for reference. As shown in Figure 8a, in the 10 mm × 10 mm × 1 mm samples, the amount of residual DNA decreased for up to 5 days of DNase treatment; however, after the amount decreased to 100–110 ng/mg-dry, it did not decrease further with additional DNase treatment. This indicated that the samples were not decellularized after exceeding the regulation value of 50 ng/mg dry weight [22,23]. 

In contrast, as shown in Figure 8b, the amount of residual DNA decreased to 35 ng/mg-dry on the first day of DNase treatment for the 710–1000 μm-sized samples. After further DNase treatment, the amount of residual DNA decreased to approximately 27 ng/mg dry weight in 5 days. This value is below the regulated value of 50 ng/mg dry weight [22,23], indicating successful decellularization. In a previous study [8], bovine cartilage decellularized by SDS and DNase treatment combined with the proteolytic enzyme elastase had a residual DNA of 10 ng/mg-wet; considering that the value in Figure 8 is based on wet weight, it was found that the amount of DNA residue could be reduced to the same level as in the previous study. As all 10 × 10 × 1 mm and 710–1000 m-sized samples that underwent only lipid extraction using liquefied DME clearly showed the presence of residual cell nuclei upon hematoxylin–eosin staining and the amount of DNA residues clearly exceeded the regulation values, these samples were not used for further detailed analysis of DNA fragment distribution.

### 3.4. DNA Fragment Distribution

**Figure 9:**
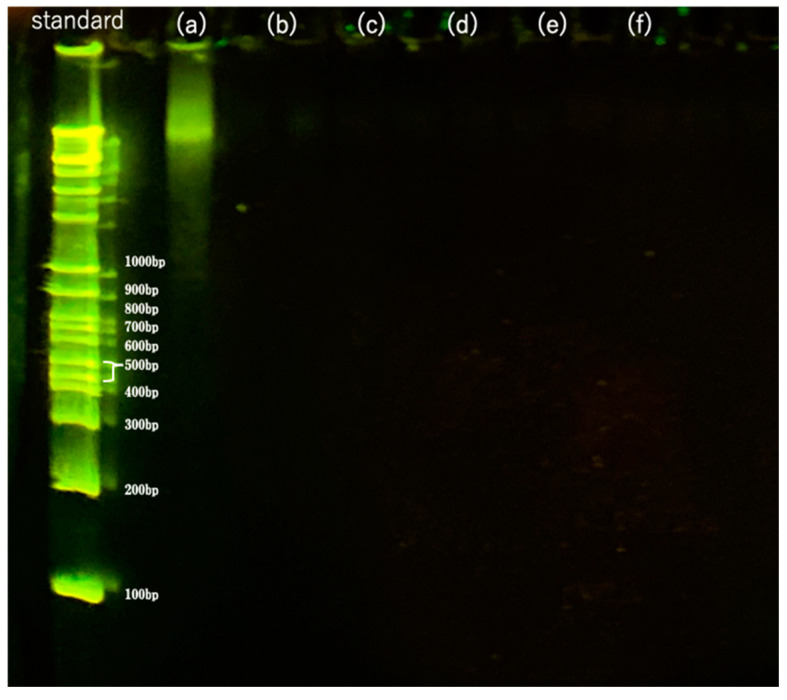
Fragments of residual DNA in the samples as detected by agarose gel electrophoresis. (**a**) Untreated. (**b**–**f**) DNase treatment for (**b**) 1, (**c**) 3, (**d**) 5, (**e**) 7, and (**f**) 10 days following DME extraction.

The results of the residual DNA fragment distribution of the 710–1000 m-sized samples decellularized with DNase for 1–10 days followed by washing are shown in Figure 9b–f. The results for the 10 × 10 × 1 mm samples are shown in Figure 9a for comparison. The leftmost lane shows the standard DNA ladder. As shown in Figure 9a, untreated samples showed mainly unfragmented DNA > 1000 bp; after treatment with DNase solution for 1–10 days, no DNA fragments were detected, as shown in Figure 9b–f, indicating that the DNA was completely degraded to <100 bp. This was below the regulation value of 200 bp [22,23], indicating that the samples were successfully decellularized. No residual DNA fragments were detected in the previous SDS treatment method [9], indicating that liquefied DME was a sufficient substitute for SDS.

Based on these results, a protocol involving lipid extraction with liquefied DME and DNA fragmentation with DNase solution, followed by washing, can be used to decellularize porcine auricular cartilage without the use of SDS. However, the efficiency depends on the sample size, and the samples need to be crushed to approximately 710–1000 μm. Despite the high permeability of liquefied DME into the tissue and sufficient lipid removal, even from the 10 × 10 × 1 mm tissue, the final decellularization was insufficient because of the very poor permeability of the aqueous DNase solution. In other words, if cartilage tissue can be processed into a disk shape, set to block the piping channels, and pressurized with an aqueous DNase solution in the direction of the tissue thickness, decellularization may be possible without pulverization.

### 3.5. FT-IR Spectra

**Figure 10:**
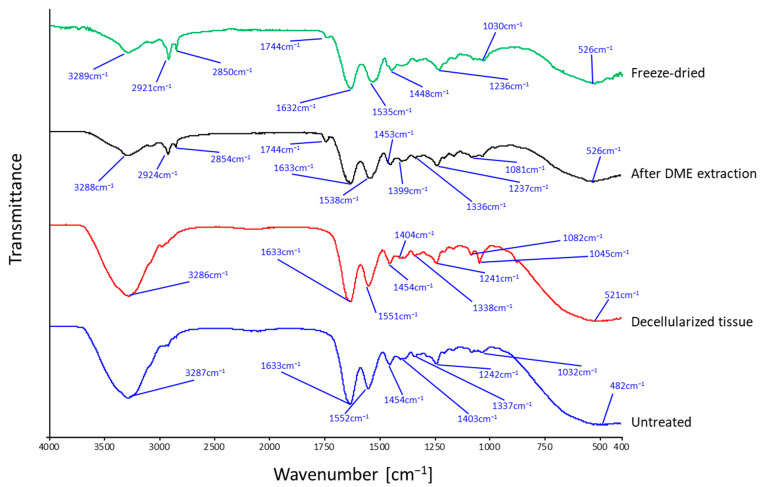
FT-IR spectra of porcine auricular cartilage.

The FT-IR spectra of the porcine auricular cartilage samples are shown in Figure 10. Porcine auricular cartilage showed nearly identical FT-IR spectra for untreated (blue curve) and decellularized (red curve) samples, but only DME-treated samples (black curve) showed a characteristic peak at 1744 cm^−1^. The peaks common to all samples were due to typical protein amide bands and were almost identical to known porcine gelatin peaks [45]. For example, the amide I band due to C=O stretching vibration at 1633 cm^−1^ and the amide II band due to C–N stretching and N–H bending vibration at 1552 cm^−1^ were typically observed [45]. In the case of untreated and decellularized porcine auricular cartilage, a large peak was observed at 3287 cm^−1^ due to the OH group of adsorbed water. In contrast, in DME-extracted porcine auricular cartilage, this large peak disappeared due to the removal of water, and the peaks at 2918 cm^−1^ and 2850 cm^−1^, which were buried due to the large peaks in the untreated and decellularized samples, appeared clearly owing to the C–H vibration common in porcine and bovine gelatins [46]. The peak at 1744 cm^−1^ observed only in the DME-extracted porcine auricular cartilage was attributed to C=N stretching vibrations caused by Schiff base reactions due to the dehydration of amino and carbonyl groups in gelatin; the same peak was also observed in gelatin with a crosslinked structure [46]. To investigate whether this change was specific to the DME extraction, the spectrum was compared with the FT-IR spectrum of freeze-dried porcine auricular cartilage (green). The FTIR spectra of the samples after DME extraction were almost identical to those of the freeze-dried samples. This implies that the observed changes were derived from the mere removal of water from the porcine auricular cartilage and were not due to any other chemical damage caused by liquefied DME. A previous study found that the history of C=N bonds produced by DME extraction impairs elasticity, but not strength, in the case of arteries; this is observed even if the C=N bonds are lost owing to the removal of the decellularized tissue [5]. In the case of the porcine auricular cartilage, lubricity is likely to be more important, thereby limiting the issue of reduced elasticity. In the future, lubricity-related measurements should be considered.

In other words, although this method can completely decellularize porcine auricular cartilage as well as the previous method, the original sample size was limited to approximately 1 mm for the decellularization of porcine auricular cartilage due to insufficient penetration of the DNase solution. Therefore, coarse grinding is necessary for pretreatment of porcine auricular cartilage. The C=N bonds temporarily generated by liquefied DME treatment are derived from a common source in the porcine aorta and cartilage, whereas the ostrich carotid artery appears to have a different chemical signature. If the chemicals responsible for this chemical feature can be identified, it may be possible to add them to porcine tissues to suppress the transient generation of C=N bonds.

## 4. Conclusions

The decellularization technique, wherein lipids are extracted with liquefied DME, followed by DNA fragmentation with DNase solution, was applied to the porcine auricular cartilage. Although the amount of DNA residue in the porcine auricular cartilage exceeded the regulatory value, it could be decellularized after treatment with DNase solution for one day using samples crushed to 710–1000 μm in size. It remains to be seen whether crushed porcine auricular cartilage can be reconstituted three-dimensionally and function adequately as a scaffold for three-dimensional culture. However, C=N bonds were formed owing to the Schiff base reaction, as previously observed in porcine aortas. How this change affects the strength of the porcine auricular cartilage is a subject for future studies. Ostrich cartilage tissue can also potentially be used in future studies, as previous studies have shown that C=N bonds do not occur in ostrich carotid arteries. The occurrence of C=N bonds was likely due to species differences. Thus, the ostrich cartilage tissue is a target for future research. The size dependence of the sample in the decellularization of porcine auricular cartilage using this technique is evident. Because of the limited permeability of the DNase solution, if an experimental setup that forces the DNase solution to flow inside the porous structure of the sample could be developed, the size-dependence problem for decellularization could possibly be solved. In another approach, if it were possible to reform crushed, but completely decellularized, cartilage tissue, the size-dependent problem could be eliminated. Nevertheless, it is essential to test the cell culture using decellularized tissue and verify its mechanical strength.

## Data Availability

Data is contained within the article.
